# Masson's Tumor of the Finger

**Published:** 2021-05-14

**Authors:** Matthew Mitchell, Charles Riccio, Muntazim Mukit, Qandeel Sadiq, Nina Krassilnik, Kalyan Dadireddy

**Affiliations:** Memphis Veterans Administration Medical Center (VAMC), Memphis, Tenn; and University of Tennessee Health Science Center (UTHSC), Memphis

**Keywords:** Masson's tumor, organizing thrombus, finger, intravascular papillary endothelial hyperplasia, hand mass

## DESCRIPTION

A 48-year-old right-hand-dominant man presented to the clinic with a history of a right index finger mass present for 2 months. He denied any trauma, drainage, or pain. On examination, the dorsoradial aspect of the proximal phalanx of the right index finger demonstrated a single, spherical, soft, nontender, freely mobile swelling that was approximately 1.5 cm long. There was no redness, punctum, or previous scars, and finger extension did not change mobility or position. The patient underwent an ultrasound scan that showed a solid vascular nodule adjacent to a vein, and he subsequently underwent excision. The mass appeared venous and had 3 contiguous vessels. Histology demonstrated a dilated blood vessel with a semiattached thrombus showing fibroblast proliferation, hemosiderin deposition, and focal pseudopapillary projection and neovascularization, consistent with Masson's tumor. The patient had an uneventful postoperative course and signed a release of information and photograph consent. This case report was deemed institutional review board exempt.

## QUESTIONS

1. What is Masson's tumor?
2. How does Masson's tumor present?
3. What are the clinical and histological features?
4. What are the treatment and prognosis?

The first description of “vegetant intravascular hemagioendothelioma,” now known as intravascular papillary endothelial hyperplasia (IPEH), was given by Masson^1^ in 1923. It is a benign lesion that accounts for approximately 2% of soft-tissue vascular tumors and is more common in females.^2^ It is a rare, benign entity, which is most commonly found on the head, neck, or upper extremities, and it typically arises within a blood vessel. However, it is generally considered to be a nonneoplastic reactive process that occurs in the context of a vascular injury.

Masson's tumor presents as a nonspecific, well-demarcated, slow-growing soft-tissue mass that most commonly manifests in the third and fourth decades of life. Clinically, Masson's tumor presents as a small (0.5-5 cm), well-defined, red or purple nodule or papule that may be located deep or superficially. These lesions tend to arise from the skin or subcutaneous tissues, but there are reports of tumors arising from the lingual mucosa, heart, and small bowel.^2^ The most common soft-tissue locations are the fingers and head and neck regions. If superficial, Masson's tumor may appear blue or red on the skin.^3^ These masses are most commonly nontender but may present with pain. Masson's tumor may be confused with angiosarcoma, vascular malformation, or Kaposi sarcoma.^4^ Ultasonography can help differentiate IPEHs from other soft-tissue lesions by detecting 1 or more associated vessels, as it did in this case.^5^ Magnetic resonance imaging findings are inconsistent, and computed tomographic scans are nonspecific. Ultimately, histology is diagnostic.^5^

Variations in IPEH histology lend itself to classification into 3 types. The primary form usually arises in the soft tissues of the hand, digits, or head and neck, oftentimes in a dilated vein. Secondary, or “mixed tumors,” arise in the presence of preexisting vascular abnormalities. The third type, extravascular IPEHs, arise in hematomas and are least common. On microscopic examination, these consist of characteristic dilated vascular channels, a fibrous connective tissue core, a monolayer of plump endothelial cells, and papillae. Tumors are generally positive for CD31, CD34, SMA, and factor VIII-related antigen. CD105 is positive on vascular endothelial cells and primary vascular neoplasms, which help differentiate IPEHs from angiosarcomas.^6^

Treatment of Masson's tumor of the skin and soft tissue consists of surgical excision. The lesion is typically well-defined from the surrounding tissue and does not require additional surgical margins.^7^ The mass may mimic malignancy on examination and imaging studies; therefore, all excised lesions should be sent for pathologic evaluation. The prognosis after excision is excellent, and recurrence is rare, usually occurring in the context of incomplete excision.^8^ Recurrence is also more likely if Masson's tumor results from an existing process, such as in type 2 or type 3 lesions. There are no consensus guidelines for frequency and length of follow-up. However, in a study cited by Katzman et al^8^ that documented recurrence, patients were followed for an average of 6.4 years, suggesting some duration of follow-up may be beneficial. Accurate diagnosis is important to prevent overaggressive resection and treatment.

## SUMMARY

Our patient presented with a nontender right index finger mass. The clinical appearance and histology supported the diagnosis of Masson's tumor. Our patient had a primary lesion, and he underwent curative surgical resection. He has not had a recurrence.

## Figures and Tables

**Figure 1:**
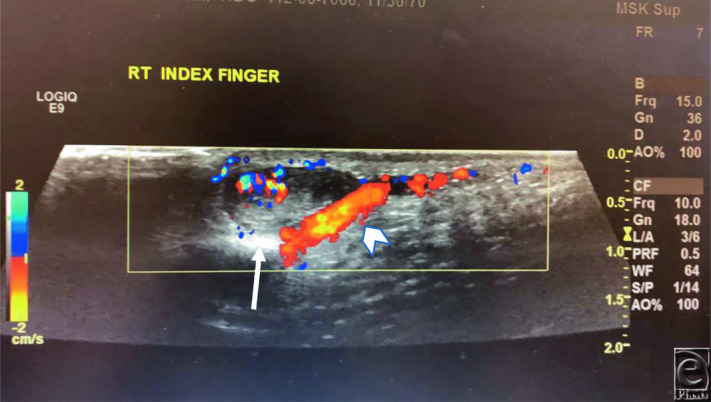
Representative right index finger ultrasound scan showing a solid vascular nodule next to a vein. White arrow points to the solid lesion, and white arrowhead points to the vein.

**Figure 2:**
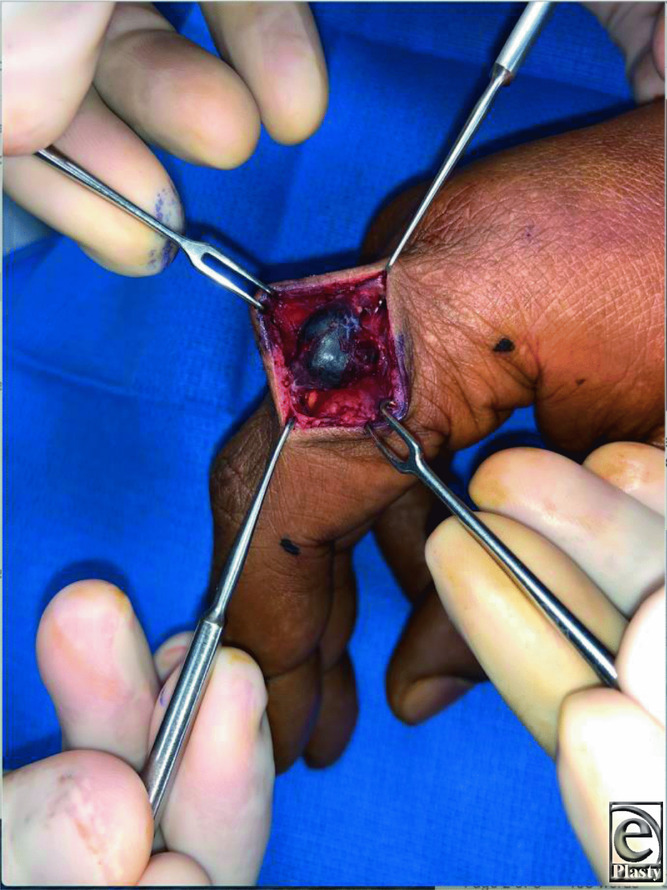
Intraoperative photograph of the index finger lesion.

**Figure 3:**
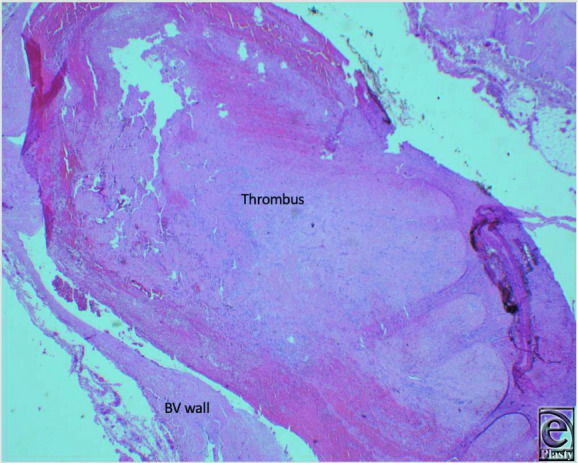
Dilated blood vessel with a semidetached organized thrombus (H&E, 2x).

**Figure 4:**
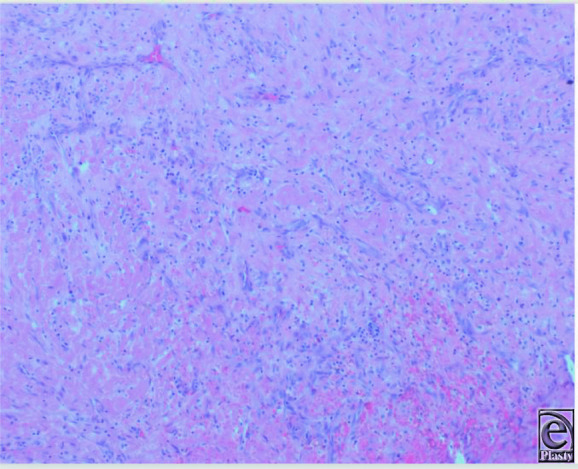
Organized thrombus with fibroblastic proliferation and neovascularization (H&E, 10x).

**Figure 5:**
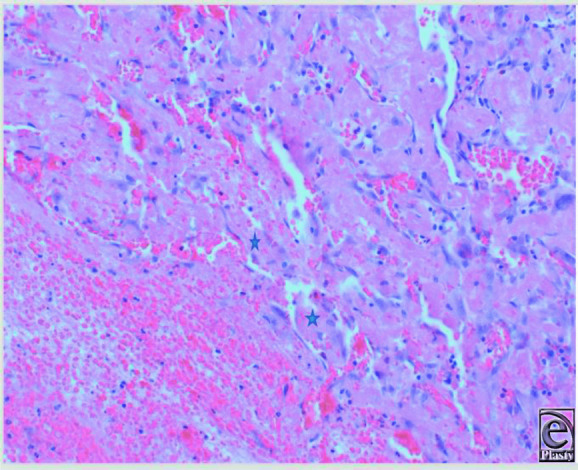
Pseudopapillary projections lined by endothelial cells (H&E, 20x).

**Figure 6:**
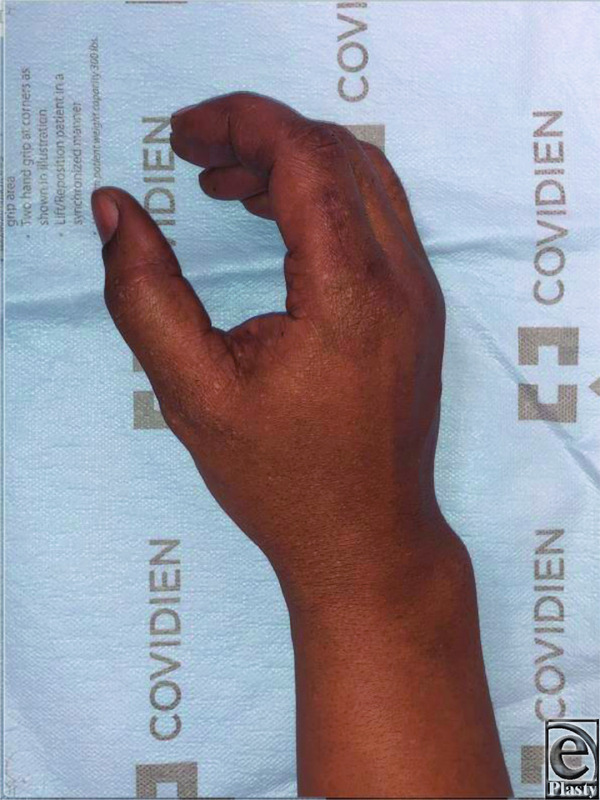
Four-month postoperative oblique photograph of the right hand showing a well-healed incision over the right index finger dorsoradial surface without recurrence.
